# Intestinal Malrotation With Chronic Midgut Volvulus, Chylous Ascites, and Severe Malnutrition in an Adolescent: A Case Report

**DOI:** 10.1002/ccr3.73496

**Published:** 2026-09-10

**Authors:** Esubalew Mihiret Alemayehu, Mulualem Amare Woldemichael, Ibrahim Hussen Kedir, Bedilu Zewdu Asmare

**Affiliations:** ^1^ Department of Pediatrics, College of Medicine and Health Sciences Dire Dawa University Dire Dawa Ethiopia; ^2^ Department of Surgery, College of Medicine and Health Sciences Wolkite University Wolkite Ethiopia; ^3^ Department of Radiology, College of Medicine and Health Sciences Wolkite University Wolkite Ethiopia

**Keywords:** adolescent, case report, chylous ascites, intestinal malrotation, midgut volvulus

## Abstract

In adolescents, chronic midgut volvulus can present insidiously over years with postprandial pain and progressive malnutrition, occasionally with chylous ascites from sustained mesenteric lymphatic obstruction. Prompt surgical correction can produce marked nutritional recovery, supporting secondary rather than primary lymphatic obstruction as the underlying mechanism.

## 1 Introduction

Intestinal malrotation arises from a failure of normal midgut rotation and fixation during embryological development. The condition most commonly declares itself in the first weeks of life with acute bilious vomiting and signs of intestinal obstruction, and roughly 90% of affected individuals are diagnosed before their first birthday [1, 2]. Beyond that window, the condition becomes considerably less common, and when it does present in older children or adults, the symptoms are typically vague enough to mislead clinicians for months or even years [3, 4, 5, 6].

In the adolescent, a chronically or intermittently twisting mesentery tends to produce a very different clinical picture from the catastrophic ischemia seen in neonates. Recurrent postprandial pain, early satiety, nonbilious vomiting, and gradual weight loss are the more typical complaints, and because none of these is specific, the diagnosis is frequently attributed to functional dyspepsia, peptic disease, gastritis, parasitic infection, abdominal tuberculosis, celiac disease, or eating disorders before the correct cause is identified [2, 3, 4, 7, 8].

When the mesenteric torsion is sustained rather than episodic, venous and lymphatic outflow can be chronically compromised, occasionally giving rise to chylous ascites and severe protein‐energy malnutrition. This combination has been reported only rarely [9, 10]. We present a 13‐year‐old boy in whom all three findings converged: a five‐year history of unrecognized chronic midgut volvulus, profound severe chronic malnutrition, and biochemically confirmed chylous ascites, ultimately corrected by a single operative intervention.

This case report has been reported in line with the SCARE (Surgical CAse REport) checklist [11].

## 2 Case History and Examination

A 13‐year‐old boy was brought to our unit with a five‐year history of dull, periumbilical pain that reliably came on after eating. His parents described repeated episodes of early satiety and nonbilious vomiting, and over time he had stopped growing normally and lost a substantial amount of weight. He had been seen at several outside facilities, where dyspepsia and parasitic infection had been treated empirically without any lasting benefit.

The parents reported no known drug allergies and no regular medication history. Review of systems was otherwise negative for fever, hematemesis, melena, hematochezia, jaundice, peripheral edema, and urinary symptoms.

There was no family history of gastrointestinal malformations, connective tissue disorders, or malignancy among first‐degree relatives. He lives with his parents and siblings in a resource‐limited rural setting, is dependent on his family for care, and had continued to attend school intermittently as his symptoms allowed; the family's diet is based on subsistence farming and locally available staples, with no other identified environmental risk factors.

On examination he was visibly cachectic. Subcutaneous fat was almost entirely absent and generalized muscle wasting was evident across all limb groups. He weighed 28 kg and measured 141 cm in height, giving a BMI of 14.1 kg/m^2^. His BMI‐for‐age z‐score was below −3 when interpreted using WHO growth references for children aged 5–19 years, meeting the WHO threshold for severe chronic malnutrition, and his mid‐upper arm circumference (MUAC) of 12 cm confirmed the degree of wasting. The abdomen was scaphoid and soft with no tenderness, guarding, or palpable organomegaly. A summary of the key clinical, laboratory, imaging, and operative findings is provided in Table 1.

**TABLE 1:** Summary of clinical, laboratory, imaging, and operative findings.

Demographics and Presentation	
Age/Sex	13 years/Male
Duration of symptoms	5 years (onset age 8)
Chief complaints	Postprandial abdominal pain, non‐bilious vomiting, early satiety
Previous diagnoses	Dyspepsia; parasitic infestation (multiple outside facilities)
Anthropometry	
Body weight	28 kg
Height	141 cm
BMI	14.1 kg/m^2^
BMI‐for‐age z‐score	< −3 (Severe Chronic Malnutrition—WHO criteria)
MUAC at admission	12 cm (severe wasting)
Laboratory findings	
Serum albumin	2.8 g/dL (hypoalbuminemia)
Hemoglobin	10.4 g/dL (mild anemia)
Liver function tests	ALT, AST, ALP, PT, PTT, INR, bilirubin — all within normal limits
Renal function tests	Within normal limits
Serum electrolytes	Within normal limits
HIV (PITC)	Negative
Peritoneal fluid triglyceride	210 mg/dL (confirms chylous ascites; threshold ≥ 110 mg/dL)
Peritoneal fluid albumin	2.0 g/dL
Peritoneal fluid WBC	350 cells/μL; lymphocytes 89%, PMN 40 cells/μL; no RBC
GeneXpert MTB/RIF (ascites)	Negative
Ascitic fluid cytology	No malignant cells
Fluid culture	Not performed (resource constraint)
CT imaging findings	
Whirlpool sign	Present — clockwise rotation of SMV and mesentery around SMA (ultrasound and CT)
SMA/SMV relationship	SMV to the left of SMA (inverted)
Duodenal position	3rd and 4th portions entirely right‐sided, failing to cross the midline
Free intraperitoneal fluid	Moderate; no CT evidence of bowel ischemia or high‐grade obstruction
Operative findings and procedure	
Degree of volvulus	360° clockwise midgut volvulus
Bowel viability	Viable throughout
Ascitic fluid volume	Approximately 400 mL of milky (chylous) fluid
Mesentery	Thickened, edematous; conspicuously dilated lymphatic channels
Ladd bands	Dense bands from cecum (epigastric) crossing duodenum
Procedure performed	Ladd procedure + appendectomy
Postoperative outcome	
1 month	Weight 30 kg (+2 kg), height 141 cm; hemoglobin 11 g/dL; MUAC 14 cm; GI symptoms resolved; tolerating normal diet
3 months (telephone)	Weight 32 kg and MUAC 14.5 cm measured by a pharmacist at nearby pharmacy; height not measured
6 months (in person)	Weight 36 kg, height 143 cm, BMI 17.6 kg/m^2^ (BMI‐for‐age z‐score ≈ −0.3, essentially normal by WHO 2007 reference); hemoglobin 12 g/dL; MUAC 15 cm; serum albumin 3.4 g/dL; returned to school; normal daily function; continuing three‐monthly outpatient follow‐up

Abbreviations: BMI, body mass index; CT, computed tomography; GI, gastrointestina; lMUAC, mid‐upper arm circumference; PITC, provider‐initiated testing and counseling; PMN, polymorphonuclear cells; SMA, superior mesenteric artery; SMV, superior mesenteric vein; WBC, white blood cell count; WHO, World Health Organization.

## 3 Differential Diagnosis, Investigations, and Treatment

**FIGURE 1:**
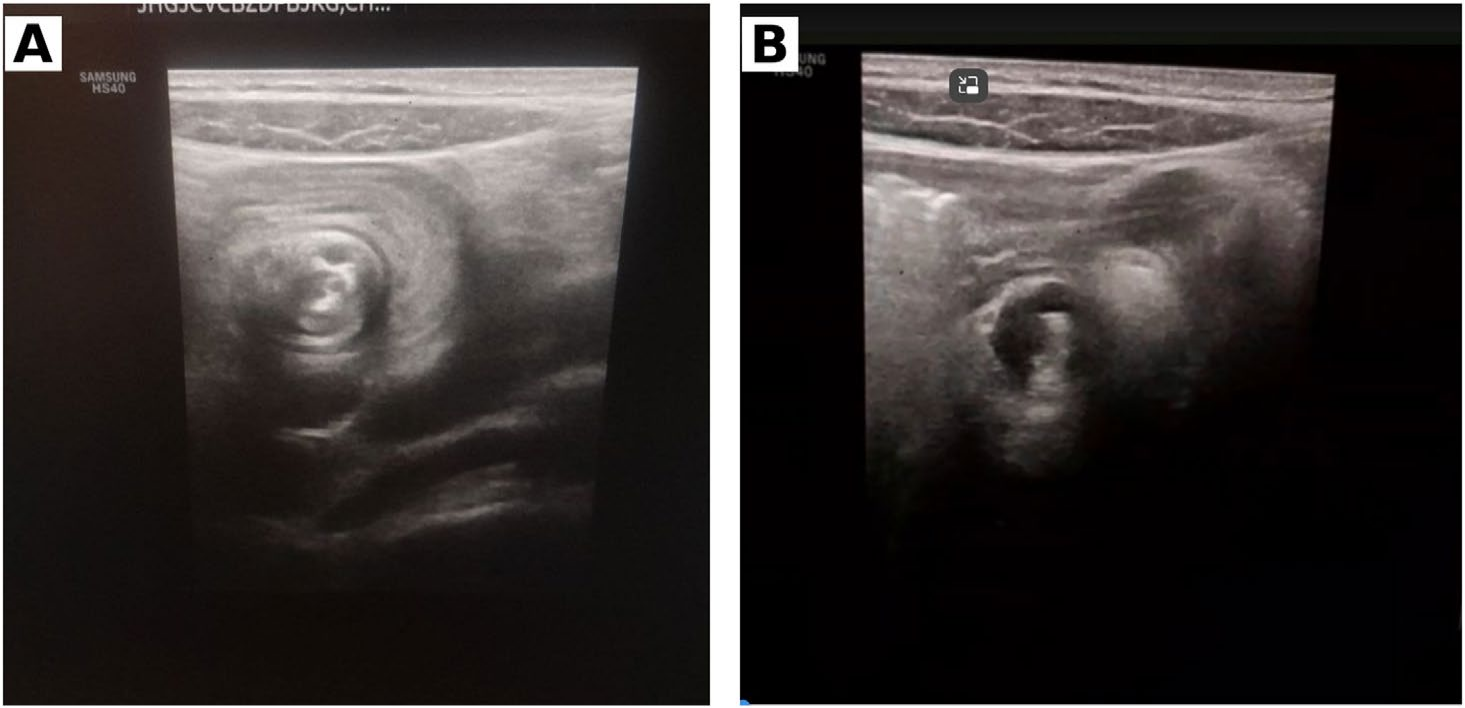
Transverse abdominal ultrasound images demonstrating the whirlpool sign. (A) Clockwise twisting of the superior mesenteric vein (SMV, arrow) around the superior mesenteric artery. (B) The SMV (arrow) and surrounding mesentery forming the characteristic swirling configuration around the SMA axis.

Admission laboratory investigations included a full blood count, liver function tests (ALT, AST, ALP, prothrombin time, partial thromboplastin time, INR, and total and direct bilirubin), renal function tests, and serum electrolytes; all were within normal limits except for a serum albumin of 2.8 g/dL (reference range 3.5–5.0 g/dL) and a hemoglobin of 10.4 g/dL (reference range 12.0–16.0 g/dL) consistent with mild anemia. HIV testing was performed by provider‐initiated testing and counseling (PITC) and returned negative. Abdominal ultrasound demonstrated the whirlpool sign [12], with clockwise twisting of the superior mesenteric vein (SMV) and mesentery around the superior mesenteric artery (SMA) (Figure 1A,B).

**FIGURE 2:**
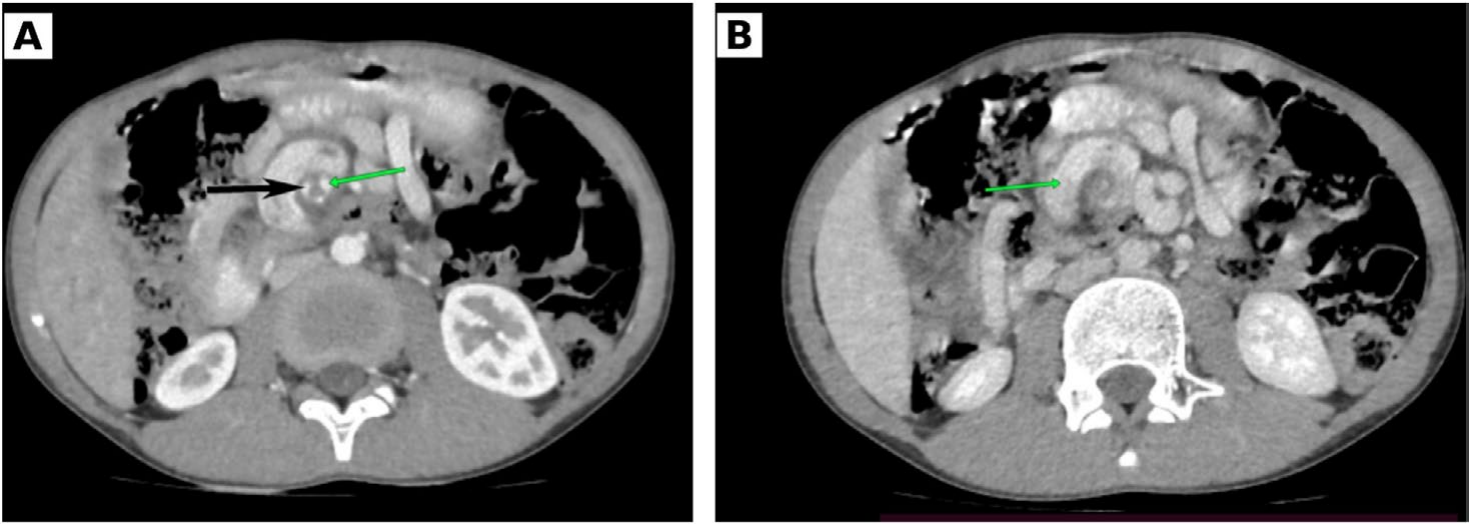
Axial contrast‐enhanced CT of the abdomen. (A) Reversal of the normal SMA–SMV relationship, with the superior mesenteric artery (SMA) to the right (black arrow) and the superior mesenteric vein (SMV) to the left (green arrow). (B) Whirling of the SMV and mesenteric fat around the SMA axis, producing the characteristic whirlpool sign.

**FIGURE 3:**
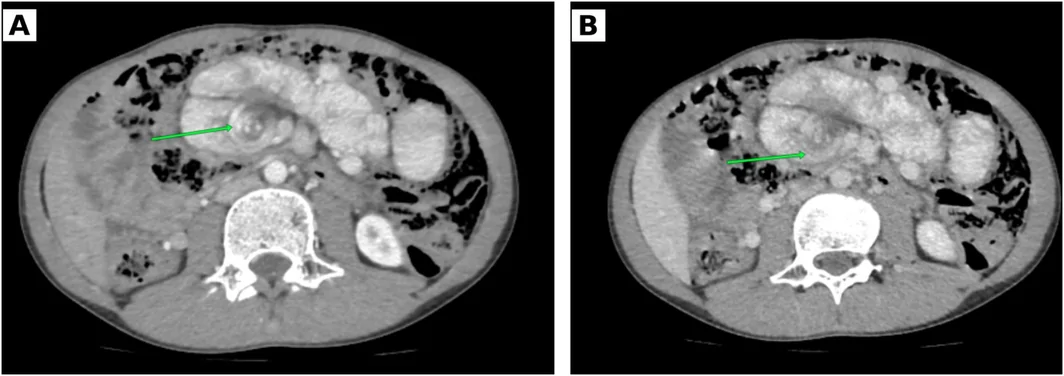
Axial post‐contrast abdominal CT scans. (A) The third part of the duodenum (arrow) failing to cross the midline. (B) The fourth part of the duodenum (arrow) also confined to the right side, with associated twisting and abnormal swirling of adjacent mesenteric fat, consistent with midgut volvulus.

Contrast‐enhanced CT of the abdomen confirmed the clockwise whirlpool configuration, with the SMV lying abnormally to the left of the SMA (Figure 2A) and whirling of the SMV and mesenteric fat around the SMA axis (Figure 2B). The third and fourth parts of the duodenum had not crossed the midline and demonstrated associated twisting, consistent with intestinal malrotation complicated by chronic midgut volvulus (Figure 3A,B). Moderate free intraperitoneal fluid was present, but there was no imaging evidence of bowel ischemia or high‐grade obstruction. Preoperative paracentesis was not performed, as the patient had no clinical signs of tense ascites, and the decision was made to proceed directly to operative management.

The differential diagnosis of chylous ascites was systematically addressed. Abdominal tuberculosis was excluded by GeneXpert MTB/RIF testing; lymphoma by cytological examination; HIV infection—epidemiologically relevant in Ethiopia [13, 14]—by PITC testing; and chronic liver disease and nephrotic syndrome by clinical assessment and liver and renal function tests. Primary intestinal lymphangiectasia was considered but is not supported by the clinical course: in a true primary lymphatic disorder, recovery on an unrestricted fat‐containing diet would not be expected, as long‐chain fatty acids would continue to be shunted into the peritoneal cavity regardless of any mechanical correction. The complete and sustained nutritional recovery on an ordinary local diet—without MCT supplementation—is most consistent with secondary lymphatic obstruction from the volvulus rather than an intrinsic lymphatic abnormality [10]. Ascitic fluid culture, celiac serology, lymphoscintigraphy, and lymphangiography were not available owing to resource constraints; these are acknowledged limitations.

After admission, fluid and electrolyte resuscitation was initiated intravenously from the point of presentation, running concurrently with the diagnostic workup. The diagnosis was confirmed within 24 h of admission. Given the finding of a 360° midgut volvulus—which carries an imminent risk of progression to full‐thickness bowel ischemia regardless of chronicity—operative intervention was undertaken on an emergency basis following rapid fluid and electrolyte optimization. Prolonged preoperative nutritional rehabilitation was not feasible within this constrained time window, as any further delay risked converting a viable bowel into an unsalvageable one. Postoperatively, the patient was initiated on a fluid diet, progressing gradually to a normal diet. Iron supplementation was started from the day of discharge and continued for 3 months. Given the resource‐limited setting, the family maintained the patient on local Ethiopian staples (injera with shiro wat), which remain the most accessible and affordable foods available. Medium‐chain triglyceride (MCT)‐based supplementation was not employed; the rationale for this decision is discussed below.

**FIGURE 4:**
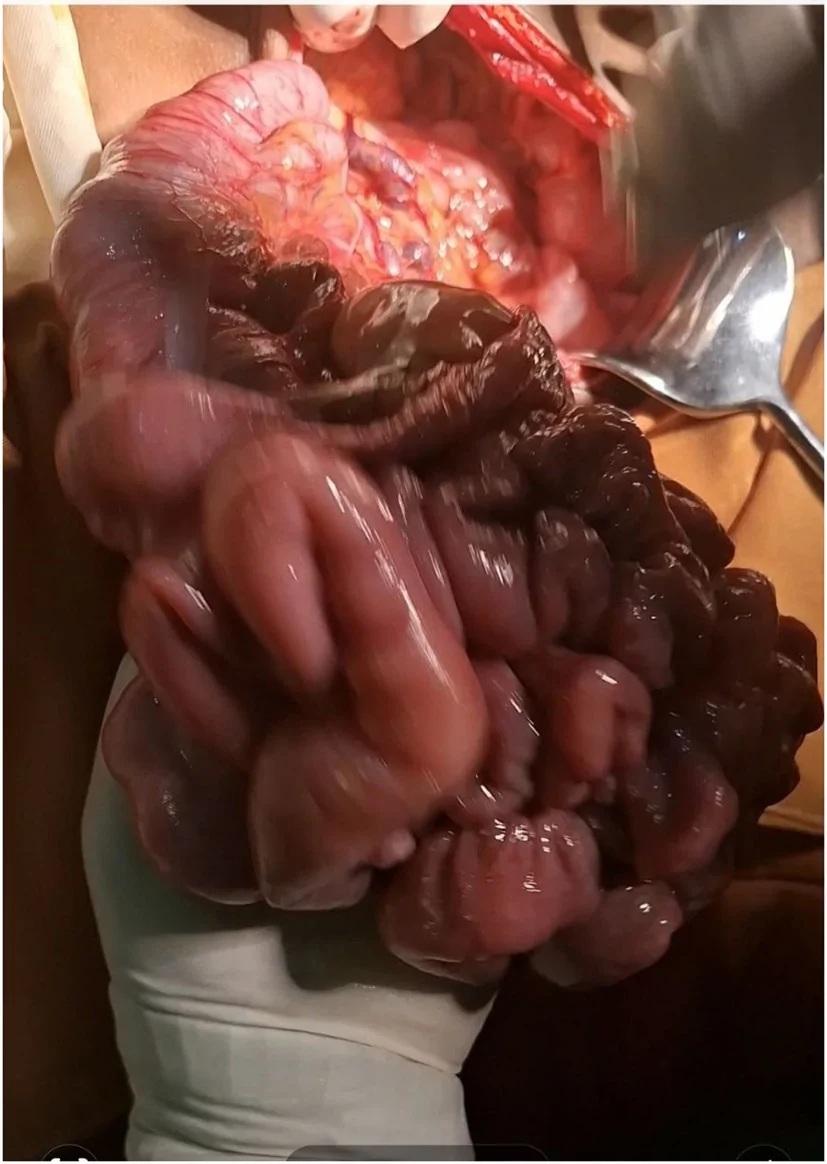
Intraoperative photograph after detorsion of the 360° clockwise midgut volvulus. The bowel is elevated to expose the mesentery. Congested, dusky loops on the right contrast with the returning pink color of the left and distal segments, reflecting hemodynamic recovery following relief of torsion; these color changes were entirely transient and resolved completely intraoperatively upon detorsion, consistent with the finding that bowel viability was preserved throughout. The mesentery is visibly thickened and edematous with conspicuously dilated lymphatic channels.

At laparotomy, a 360° clockwise volvulus of the midgut was found around a markedly narrowed mesenteric pedicle. Approximately 400 mL of milky fluid was present in the peritoneal cavity; analysis returned a triglyceride level of 210 mg/dL, confirming the diagnosis of chylous ascites. Ascitic fluid albumin was 2.0 g/dL. Additional fluid analysis showed a white cell count of 350 cells/μL with a lymphocyte predominance (89%; absolute polymorphonuclear cell count 40 cells/μL) and no red blood cells. GeneXpert MTB/RIF testing of the ascitic fluid was negative, and cytological examination revealed no malignant cells. Fluid culture could not be performed owing to resource constraints. The bowel itself remained viable throughout; transient dusky discoloration of some bowel loops observed at the time of laparotomy resolved completely upon detorsion, confirming preserved viability. The mesentery was thickened and edematous with conspicuously dilated lymphatic channels (Figure 4). The cecum lay in the epigastrium, tethered by dense Ladd bands that compressed the duodenum.

A standard Ladd procedure was carried out: the bowel was detorsed counterclockwise, the Ladd bands were divided, the mesenteric pedicle was widened, and the small bowel was repositioned to the right with the colon to the left. Appendectomy was performed concurrently, as is standard practice with the Ladd procedure, to eliminate any future risk of diagnostic confusion given the abnormal anatomical position of the appendix. The appendix was macroscopically normal and, consistent with local institutional practice in a resource‐limited setting, was not submitted for routine histopathological examination. The patient recovered without complication.

The procedure was performed by a consultant pediatric surgeon (MAW) with extensive experience in pediatric general and gastrointestinal surgery at a university‐affiliated referral hospital where the pediatric surgical service manages intestinal malrotation and midgut volvulus as part of its routine caseload.

## 4 Conclusion and Results (Outcome and Follow‐Up)

Postoperative progress was encouraging and followed a steady upward trajectory. At 1 month the patient had gained 2 kg (weight 30 kg) and his MUAC had risen to 14 cm. He was tolerating a normal diet and his gastrointestinal symptoms had resolved. Subsequent follow‐up was conducted by telephone at 3 months owing to difficulties with in‐person attendance; his weight was reported as 32 kg and MUAC as 14.5 cm (measured by a pharmacist at a nearby pharmacy and communicated by phone—values that should be interpreted with caution given the non‐standardized measurement conditions). At the six‐month in‐person review, weight was 36 kg and height 143 cm, giving a BMI of 17.6 kg/m^2^. Using the WHO 2007 growth reference for children aged 5–19 years, this corresponds to a BMI‐for‐age z‐score of approximately −0.3—essentially within the normal range and a striking contrast to the admission z‐score of below −3. The 2‐cm height gain observed over the six‐month follow‐up period is consistent with early catch‐up linear growth, a recognized phenomenon during nutritional recovery in previously malnourished children and adolescents. His mother reported that he had returned to school and was eating and functioning normally. He remains under active follow‐up on three‐monthly outpatient appointments, with ongoing nutritional monitoring and iron supplementation continuing until all anthropometric and biochemical markers fully normalize. (Table 2).

**TABLE 2:** Case Timeline.

Time Point	Event
Age 8 years	Onset of intermittent postprandial abdominal pain and non‐bilious vomiting.
Age 8–13 years	Multiple evaluations at outside facilities. Empirical treatment for dyspepsia and parasitic disease without improvement. Progressive weight loss and growth failure.
Age 13 — admission	Referral and admission. Severe chronic malnutrition confirmed (BMI 14.1 kg/m^2^, MUAC 12 cm, albumin 2.8 g/dL). Comprehensive initial workup (LFTs, RFTs, electrolytes, HIV PITC) unremarkable except for hypoalbuminemia and mild anemia.
Admission—day 1	Abdominal ultrasound and contrast‐enhanced CT performed and confirmed intestinal malrotation with chronic midgut volvulus. Rapid IV fluid and electrolyte resuscitation initiated. Emergency operative intervention undertaken on the same day given imminent risk of bowel ischemia from 360° volvulus.
Surgery	Exploratory laparotomy: 360° clockwise midgut volvulus confirmed. Approximately 400 mL of chylous ascites (triglyceride 210 mg/dL, albumin 2.0 g/dL). Ladd procedure with appendectomy performed. Bowel viable throughout.
1 month postoperative	In‐person review: weight 30 kg (+2 kg), height 141 cm; hemoglobin 11 g/dL; MUAC 14 cm. Complete symptom resolution. Tolerating a normal diet.
3 months postoperative (telephone)	Telephone follow‐up: weight 32 kg and MUAC 14.5 cm measured by a pharmacist at nearby pharmacy communicated and instructed by phone. Height was not measured.
6 months postoperative (in person)	In‐person follow‐up: weight 36 kg (+8 kg from admission), height 143 cm; BMI 17.6 kg/m^2^ (BMI‐for‐age z‐score≈ −0.3, essentially within normal range by WHO 2007 reference); hemoglobin 12 g/dL; MUAC 15 cm; serum albumin 3.4 g/dL. Height gain of 2 cm from admission consistent with early catch‐up linear growth. Returned to school with normal eating and daily activities. Continuing three‐monthly outpatient follow‐up.

### 4.1 Patient/Family Perspective

The patient's father stated, “I could not imagine he would become healthy like this again. This recovery feels like a miracle for our whole family, not only for my son.” The family expressed great satisfaction with the outcome and the patient's improvement in health and nutritional status.

## 5 Discussion

Intestinal malrotation with chronic midgut volvulus can present atypically in adolescents, with years of nonspecific symptoms preceding the diagnosis [1, 2, 5]. Chylous ascites and severe malnutrition may develop as complications of sustained mesenteric lymphatic obstruction. Peritoneal fluid triglyceride measurement is essential to confirm chylous ascites and guide the differential diagnosis [9, 10]. Prompt surgical correction can reverse even severe nutritional compromise, and recovery on an unrestricted diet is most consistent with a secondary rather than a primary lymphatic etiology. Clinicians should maintain a low threshold for cross‐sectional imaging in adolescents with progressive weight loss and meal‐related abdominal symptoms [4, 6, 8, 12, 15].

This case illustrates three features of educational importance: a five‐year diagnostic delay before the correct diagnosis was reached, the unusual coexistence of chronic midgut volvulus with chylous ascites and severe malnutrition, and marked nutritional recovery following surgical correction alone.

Malrotation is predominantly a disease of the newborn period, yet delayed presentation in older children and adolescents is well documented [1, 2, 3, 5]. Durkin and colleagues showed that patients diagnosed outside the neonatal period tend to have experienced longer diagnostic journeys and a different spectrum of morbidity [3]. When volvulus is partial or intermittent, sufficient arterial inflow may be preserved to maintain bowel viability while venous and lymphatic return remains chronically impaired, producing a gradual clinical deterioration over months or years rather than an acute surgical emergency [2, 4, 8].

A five‐year diagnostic delay of this kind, while unfortunate, is not unusual in adolescents presenting with chronic nonspecific abdominal symptoms [3, 6, 7]. These patients are typically investigated for functional disorders, gastritis, or parasitic disease, and cross‐sectional imaging is often deferred until clinical deterioration is advanced [3, 6, 7]. In a child with progressive weight loss and meal‐related symptoms, the threshold for CT or upper gastrointestinal contrast imaging should be low [2, 7]. Kabeer and colleagues noted that the chronicity of symptoms in older patients sometimes prompts debate about whether surgery is necessary; their conclusion, with which we agree, is that symptomatic malrotation at any age warrants operative correction [6].

The coexistence of chylous ascites with chronic midgut volvulus is uncommon; only a small number of cases in older children have been described [7, 9]. Mechanistically, sustained torsion at the mesenteric root obstructs lymphatic drainage; the resulting rise in lymphatic pressure causes chyle to leak into the peritoneal cavity. Concurrent malabsorption from a chronically obstructed gut compounds nutritional losses, and the combination is sufficient to produce severe protein‐energy malnutrition even in the absence of bowel ischemia. The serum‐to‐ascites albumin gradient (SAAG) of 0.8 g/dL in this patient is consistent with a non‐portal hypertensive etiology such as lymphatic obstruction, further supporting this mechanism [10].

Upper gastrointestinal contrast series is the traditional reference standard for diagnosing malrotation in most pediatric centers [8], but was not available at our institution. Contrast‐enhanced CT provided comprehensive assessment of mesenteric vessel orientation, bowel position, and viability, and reliably demonstrated the whirlpool sign and failure of duodenal fixation [4, 8, 12].

Once midgut volvulus was confirmed, operative correction was undertaken without delay. The American Pediatric Surgical Association supports surgery for symptomatic malrotation regardless of patient age [2, 15], and deferral was not considered appropriate given the 360° torsion and imminent risk of ischemia. The nutritional recovery was notable: the patient gained 8 kg over 6 months on an unrestricted local diet, reaching a BMI‐for‐age z‐score of approximately −0.3—a near‐complete normalization from the admission value of below −3. The progressive weight gain alongside resolution of gastrointestinal symptoms strongly suggests that nutritional failure was driven by the mechanical and lymphatic obstruction rather than by intrinsic gut pathology [2, 7, 15].

### 5.1 Limitations

Several limitations should be acknowledged. Ascitic fluid culture, celiac serology, lymphoscintigraphy, and lymphangiography were unavailable owing to resource constraints. The three‐month follow‐up measurements were obtained by telephone and recorded non‐standardly; these values should be interpreted with caution. The SAAG calculation carries a minor temporal limitation as serum and ascitic albumin were not collected simultaneously. These constraints reflect the realities of practice in a resource‐limited setting but are not considered to substantially alter the clinical conclusions.

## Author Contributions

**Esubalew Mihiret Alemayehu:** conceptualization, writing – review and editing, data curation. **Mulualem Amare Woldemichael:** writing – original draft, validation. **Ibrahim Hussen Kedir:** investigation. **Bedilu Zewdu Asmare:** investigation.

## Funding

The authors have nothing to report.

## Disclosure

Reporting Guideline Compliance: This case report has been prepared and reported in accordance with the SCARE (Surgical CAse REport) 2023 guidelines [11].

## Consent

Written informed consent was obtained from the patient's parent/legal guardian for the publication of this case report, including all clinical details, imaging findings, and intraoperative photographs. Verbal assent was also obtained from the patient. Documentation of both consent and assent is available upon request by the Editor.

## Conflicts of Interest

The authors declare no conflicts of interest.

## Data Availability

All data supporting the findings of this case report are included within the manuscript. The signed patient/guardian consent and assent documentation are not published but are available from the corresponding author upon reasonable request, in line with the consent statement above.
